# Understanding Racial Disparities in Exposure to Traffic-Related Air Pollution: Considering the Spatiotemporal Dynamics of Population Distribution

**DOI:** 10.3390/ijerph17030908

**Published:** 2020-02-01

**Authors:** Yoo Min Park, Mei-Po Kwan

**Affiliations:** 1 Department of Geography, Planning, and Environment, East Carolina University, Brewster Building A-237, MC-557, Greenville, NC 27858, USA; 2 Department of Geography and Resource Management, The Chinese University of Hong Kong, Shatin, Hong Kong 999077, China; mpk654@gmail.com; 3 Institute of Space and Earth Information Science, The Chinese University of Hong Kong, Shatin, Hong Kong 999077, China; 4 Department of Human Geography and Spatial Planning, Utrecht University, 3584 CB Utrecht, The Netherlands

**Keywords:** traffic-related air pollution, exposure to PM_2.5_, multi-contextual segregation, environmental health disparities, spatiotemporal methods, human mobility, environmental justice, uncertain geographic context problem, neighborhood effect averaging problem

## Abstract

This study investigates the effect of spatiotemporal distributions of racial groups on disparities in exposure to traffic-related air pollution by considering people’s daily movement patterns. Due to human mobility, a residential neighborhood does not fully represent the true geographic context in which people experience racial segregation and unequal exposure to air pollution. Using travel-activity survey data containing individuals’ activity locations and time spent at each location, this study measures segregation levels that an individual might experience during the daytime and nighttime, estimates personal exposure by integrating hourly pollution maps and the survey data, and examines the association between daytime/nighttime segregation and exposure levels. The proximity of each activity location to major roads is also evaluated to further examine the unequal exposure. The results reveal that people are more integrated for work in high-traffic areas, which contributes to similarly high levels of exposure for all racial groups during the daytime. However, white people benefit from living in suburbs/exurbs away from busy roads. The finding suggests that policies for building an extensive and equitable public transit system should be implemented together with the policies for residential mixes among racial groups to reduce everyone’s exposure to traffic-related air pollution and achieve environmental justice.

## 1. Introduction

Numerous studies have found that the burden of air pollution is disproportionately distributed among racial/ethnic groups. Many of these studies examined the role of residential segregation in understanding the disparities in exposure to air pollution [1,2,3]. However, a growing body of literature has suggested that people may experience racial segregation and exposure to air pollution beyond their residential contexts because they move through multiple microenvironments to undertake daily activities [4,5,6]. It has been revealed that racial/ethnic groups do not necessarily work and play together in the same part of an urban area [7,8]. Despite the advances in infrastructure and technology and rapid increase in automobile use in modern societies, different population groups do not travel to the same extent or with the same resources because their mobility patterns are often determined by a variety of social, financial, political, and cultural factors. This indicates that segregation among different population groups could take place at any time and any place due to variation in mobility patterns. 

To articulate the full spectrum of segregation that occurs in multiple places throughout the day, Park and Kwan [5] proposed a new comprehensive term, “multi-contextual segregation”. Multiple contexts in this term include not only spatial contexts of daily life (e.g., home, work, and social/recreational places) but also temporal contexts in which an individual is situated (e.g., times of day). By developing and utilizing an index that measures multi-contextual segregation, they found that levels of racial segregation were lower during the daytime (when many people spend their time on working or conducting activities outside of their home) than at night (when most people stay at home) in the Atlanta metropolitan area, Georgia, U.S. However, even during the daytime, the inner city or inner-ring suburbs remained relatively highly segregated. This finding implies that the association between segregation and racial disparities in exposure to air pollution may vary by time. Such dynamic, multi-contextual segregation and people’s daily mobility complicate efforts to understand the link between segregation and environmental disparities.

The effect of multi-contextual segregation on environmental health disparities has been relatively less studied to date when compared to that of residential segregation. Limiting the geographic context to a residential neighborhood in the analysis by using residence-based, aggregate-level data has led to the discrepancy in findings on the association between segregation and unequal exposure to air pollution [9]. This may be because the narrow focus causes fundamental methodological problems that may have made the previous results less reliable, including the uncertain geographic context problem (UGCoP)––a problem that arises when the true geographic context in which people are exposed to social and environmental factors is mis-delineated [10] and the modifiable areal unit problem (MAUP)––a problem that research results may vary depending on which areal (or aggregated) unit is used [11].

Spatiotemporal variability in air pollution concentrations adds another layer of complexity to examining environmental inequalities associated with exposure to air pollution. Air pollution concentrations significantly vary across space and continuously change over time, even for a short period [12]. Due to its high variability, deploying high-resolution air pollution data is essential to environmental justice research on exposure to air pollution. Compared to any other pollution sources, traffic emissions often cause dramatically high spikes in pollution concentrations near roadways [13]. A disproportionate number of racial minorities tend to live near major roads compared to the majority group [14,15,16]. This may place them at an unequally high health risk because air pollution specifically from vehicle emissions has profound impacts on human health. In particular, particulate matter with a diameter of less than 2.5 micrometers (PM_2.5_) generated from road traffic can cause several adverse health problems even at very low concentrations because it can penetrate deeply into the lung (WHO, 2014). Several studies suggested that this disparity was greater by race and ethnicity than by income levels, educational attainment, or other indicators of socioeconomic status [16,17].

In light of the above discussion, this study highlights that the scope of environmental justice research should be extended to include the full spectrum of geographic contexts in which people are exposed to environmental health hazards and experience segregation. What remains understudied is how multi-contextual segregation influences the racial inequality in exposure to air pollution, particularly PM_2.5_ generated from on-road mobile sources. The literature offers relatively little on how the differences in locations of workplaces, schools, and other places in which different racial groups spend their time influence the racial disparities in health risks associated with exposure to traffic-related PM_2.5_. Therefore, this study aims to investigate how nighttime segregation (closely related to residential segregation) and daytime segregation (closely related to non-residential segregation) are associated with exposure to traffic-related PM_2.5_. It uses individual-level data for multi-contextual segregation and individual estimates for exposure to traffic-related PM_2.5_. It hypothesizes that the associations differ by racial group and at different times of day: for white people, greater daytime and nighttime segregation are associated with lower levels of exposure to traffic-related PM_2.5_, while the reverse is true for people of color. In this study, the following questions are answered: (1) Which racial group faces the highest/lowest level of exposure to traffic-related PM_2.5_? (2) How does segregation at different times of day differentially affect exposure to traffic-related PM_2.5_ among racial groups? and (3) Is the percentage of African-Americans who spend time near major roads higher than that of white people in all time periods of a day?

## 2. Materials and Methods 

**Figure 1:**
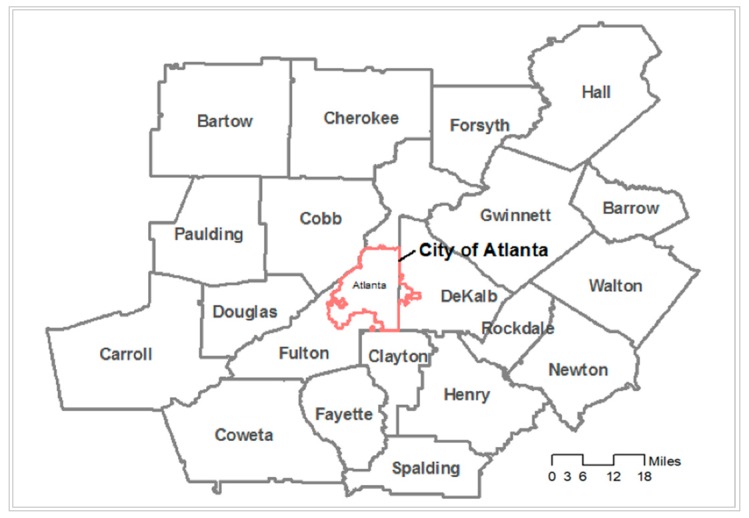
The study area (the Atlanta metropolitan area).

The study area is the Atlanta metropolitan area (Georgia, U.S.) that includes 20 counties (Figure 1). As one of the major metropolitan areas in the U.S., the region is characterized by a large African-American population, suburbanization of jobs and of whites and the middle class, sprawl-related long commutes, a high level of automobile reliance, and serious traffic congestion that aggravates air pollution [18]. Due to the high traffic, this region is one of the most polluted U.S. metropolitan areas [19]. Atlanta has also been reported as the second most segregated city [20] because of racial discrimination in housing and poor public transportation. These characteristics offer an important context for examining how racial segregation contributes to the disparity in exposure to traffic-related air pollution. 

Multi-contextual segregation is measured using the individual-level spatiotemporal proximity index (*i*-STP index) [5]. The *i*-STP index, a modified version of Grannis’s [21] residential segregation index, measures the relative spatial proximity of activity locations among multiple groups during a specific time using an inverse distance function. The level of segregation that each individual experiences in a given period is evaluated within a person-specific neighborhood defined by *k*-nearest neighbors from the individual’s activity location. There are several advantages of this index over Grannis’s index and other similar residential segregation indices (e.g., White’s [22] spatial proximity index). First, it is an individual-level index that uses the exact activity locations at which an individual stayed, unlike Grannis’s index that often relies on centroids of spatial units (e.g., census tract) to represent individuals’ residential locations [5]. Therefore, it is less subject to aggregation errors that arise from aggregating point data into areal units [23]. It can also measure segregation levels not only at an individual’s residential location but also at his/her various other activity locations [5]. In addition, while Grannis’s index produces a global value for the entire region using the total population, the *i*-STP index calculates a local index for each individual by only including *k* number of spatiotemporally close neighbors of the individual in the calculation. In this study, we define *k* as 200 (i.e., 200-nearest neighbors whose activity duration overlaps with that of the individual), taking the spatial distribution of the respondents into account, and use a 7-km distance threshold (*d*) to allow an individual in areas with low population density to have less than 200 neighbors if they are not found within the distance. More detailed descriptions of the index, formulation, and choices of *k* and *d* parameters can be found in Park and Kwan [5].

The index value of 1.0 means racial integration during the given time. If it is higher than 1.0, it indicates that an individual is likely to experience racial segregation within the person’s spatiotemporal neighborhood. Using the *i*-STP index, we create two segregation variables to characterize spatial distributions of racial groups during the daytime and at night. We define daytime segregation as segregation between 6 a.m. and 6 p.m. because daytime is defined as after sunrise (6 a.m.) and before sunset (6 p.m.) in general. Each individual thus has two segregation index values. Individuals’ activity locations and racial/ethnic information are obtained from travel-activity diaries in the Atlanta Regional Commission (ARC) Reginal Travel Survey (2011). The travel-activity diaries include 25,810 individuals’ geocoded activity locations, start and end times for each activity, activity types, as well as their personal characteristics, including race, ethnicity, gender, age, household income, and educational attainment (non-Hispanic whites (51.0%), African Americans (31.1%), Hispanics (10.9%), and Others (i.e., Asian, Native American, Alaskan Native, Pacific Islander, and Native Hawaiian) (7.0%)). This data is remotely accessed via the secure portal of the Transportation Secure Data Center in the National Renewable Energy Laboratory. All data analyses using this data were conducted with the approval of the Institutional Review Board (IRB) from the Office for the Protection of Research Subjects of the University of Illinois at Urbana-Champaign (IRB#: 17853).

**Figure 2:**
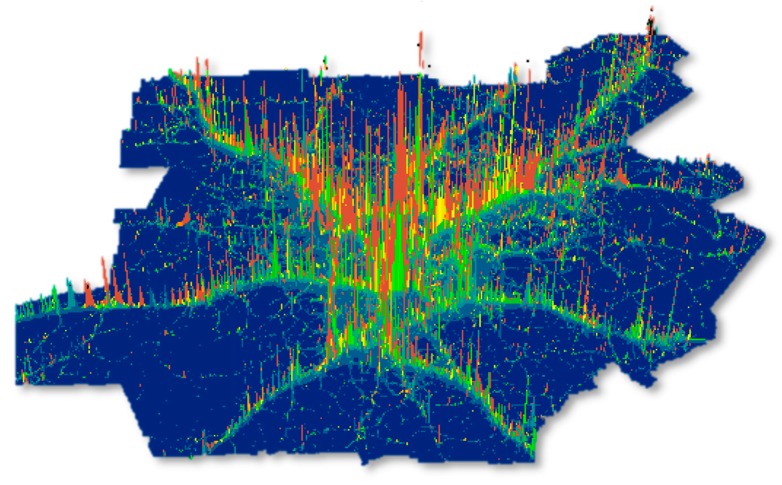
3D geovisualization of traffic-related particulate matter (PM_2.5_) concentrations (R-LINE model output).

An individual’s daily exposure level is estimated by extracting a PM_2.5_ concentration at each location in which the person spent time from an hourly pollution map at the corresponding hour using geographic information system (GIS) techniques and then, averaging the concentrations based on the time spent at each location. We use travel-activity diaries to identify all locations visited by each individual and air dispersion model outputs to characterize hourly PM_2.5_ concentrations. Because the travel-activity diaries include only the locations in which the respondents stayed for a certain amount of time for conducting daily activities, travel routes from an activity location to another are estimated using the geospatial method developed by Park [24]. This method estimates a probable travel route between two activity locations using the Google Maps Directions API and considering the travel modes that the respondents actually used and travel times they reported. The traffic-related PM_2.5_ concentrations are modeled using R-LINE, a line-type source air pollution dispersion model developed by the US EPA Office of Research and Development [25,26,27]. R-LINE is designed to capture the fine-scale local variation of roadway air pollution concentrations by considering traffic emissions on road segments and meteorological conditions [26]. It requires emission data, meteorological data, and a reception grid as inputs. We obtain roadway link-based emission data from the Atlanta Regional Commission [28], which includes the annual average hourly PM_2.5_ emissions for all major and small roads in the Atlanta metropolitan area and information about link segments (e.g., each link segment’s length and georeferenced start and end points). We also obtain pre-processed hourly meteorological data from Lakes Environmental Consultants Inc. and create a 200-meter receptor grid across the modeling area. A concentration at each receptor is then calculated using the emission and meteorological data in the R-LINE software. The concentration maps are created based on the model outputs using ESRI’s ArcGIS software (Version 10.6). Figure 2 shows that areas near major roads in the inner cities and inner-ring suburbs (colored in red) have high concentrations. 

To answer the first research question, an analysis of variance (ANOVA) is used to compare the average daily exposure levels among racial groups. The Tukey’s honest significant difference test is then conducted to examine between which racial groups the differences occur and by how much they differ. These group-level average exposure estimates are more reliable than those derived from residence-based methods because the uncertainty of the geographic context in which people are exposed to air pollution is mitigated due to the use of individual movement data, and thus they are less affected by the UGCoP.

Next, multiple linear regression models are used to investigate the differential effect of nighttime and daytime segregation on total daily exposure to traffic-related PM_2.5_. Regression is run separately for each racial group to see racial differences in the segregation effect. In the model, personal daily exposure levels are used as a dependent variable, and nighttime and daytime segregation levels are used as the main independent variables. The regression model also includes other individual characteristics, such as gender, age, household income, and educational attainment, as control variables. Gender, household income, and educational attainment are dummy coded with males, individuals with household income higher than $59,999, or individuals with undergraduate or graduate degrees, as reference groups (Gender = 1 if females; Income dummy 1 = 1 if household income ≤ $29,999; Income dummy 2 = 1 if household income > $29,999 and ≤ $59,999; Educational dummy 1 = 1 if not a high school graduate, 12 grade or less; Education dummy 2 = 1 if high school graduate, some college credit but no degree, or associate/technical school degree). Various assumptions of multiple regression analysis are tested using diagnostic plots, including QQ plots for the normality assumption and scatterplots for the assumption of the constant variance of residuals. 

Lastly, buffers are created within 200 meters from the major roads (i.e., roads with > 3 lanes) to answer the third research question. Many previous studies assessing exposure to traffic-related air pollution and its health risk performed buffer analysis to use the residential proximity to major roads as a proxy for exposure to traffic-related air pollution [29,30,31]. This study goes beyond the residence-based proximity models and measures the proximity of an individual to roadways from all daily activity locations. The 200-m buffer size is determined based on previous findings that PM_2.5_ levels decrease to near background concentrations at distances of 100–200 meters from major roads [13]. The 200-m buffers are overlaid with people’s daily activity locations at different times of day to examine if the percentage of African-Americans near major roadways in each time period of a day is higher than that of white people. 

## 3. Results

The ANOVA result suggests that exposure levels of traffic-related PM_2.5_ (*p* < 0.001) are significantly different among racial/ethnic groups. White people have the lowest average exposure levels at 1.1841 μg/m^3^, followed by Hispanics at 1.2404 μg/m^3^, African-Americans at 1.3345 μg/m^3^, and Others at 1.3477 μg/m^3^. The result of the Tukey’s test (Table 1) indicates that the difference of means is statistically significant between white people and African-Americans (*p* < 0.001), white people and Others (*p* < 0.001), and Hispanics and African-Americans (*p* = 0.0306).

**Figure 3:**
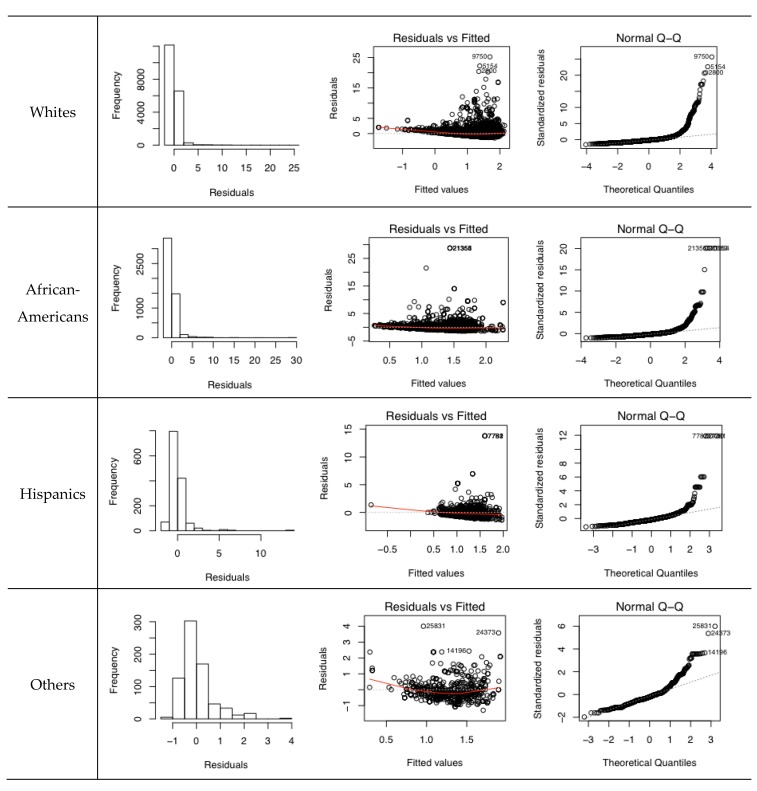
Diagnostic plots of multiple linear regression models.

Regression analyses are conducted to examine the effect of multi-contextual segregation on the differential levels of exposure to traffic-related PM_2.5_ among racial groups. Table 2 shows the descriptive statistics for the dependent and independent variables. To check if residuals of the regression models are normally distributed, histograms of residuals for each regression model are created. The residuals of all regression models have strongly positively skewed distributions (Figure 3). The diagnostic plots also suggest that the normality assumption may not be valid in all four regression models because the Q–Q plots do not present straight lines. In addition, the plots of residuals versus predicted values indicate that the assumption of constant variance is not likely to be true, because a clear funnel shape is observed in all four plots. To make the dependent variable and main independent variables normally distributed and achieve approximate homoscedasticity, this study log-transforms these variables and re-runs the regression models.

Table 3 shows that white people’s roadway exposure is negatively associated with both nighttime (*p* < 0.001) and daytime segregation (*p* < 0.001). This means that the greater the segregation—which white people experience at higher levels at residential locations, in the workplaces, and at other daily activity places—the less PM_2.5_ concentrations to which they are exposed. In general, people in power have the social privilege and the financial capacity (e.g., car ownership) to select their activity locations, including homes and workplaces. White people tend to be willing to pay commuting costs in order to live in white-flight neighborhoods that may be far from their workplaces [32]. This negative association can also be interpreted as indicating that if white people are more integrated with other racial groups, they are more likely to be exposed to higher traffic-related PM_2.5_. This effect is stronger during the daytime than at night, which may be because traffic-related PM_2.5_ concentrations are high in the central business district in which white people and other racial groups work together during the daytime.

The negative association between daytime segregation and exposure to traffic-related PM_2.5_ is also significant for African-Americans, Hispanics, and Others. Again, the result demonstrates that people are more likely to experience similarly high levels of exposure to traffic-generated PM_2.5_, regardless of their race/ethnicity if they are less segregated for work during the daytime. This result is an empirical example of the neighborhood effect averaging problem (NEAP) [33]. The NEAP refers to the problem that the neighborhood effect on environmental exposure may be over- or underestimated if it is assessed with a residence-based approach and human mobility is ignored. 

This study then identifies the geographic locations of individuals whose daytime segregation index values are between 1.0 (no racial segregation) and 1.2 (low level of segregation). It finds that the areas in which people experience more racial integration during the daytime present higher traffic-related PM_2.5_ concentrations than the other areas in the metro Atlanta region do. The racial integration in the central cities during the daytime may simply be because of the influx of white suburban/exurban commuters. In contrast, no recognizable racial integration is observed in large job centers in the suburbs––where roadway PM_2.5_ concentrations are relatively low. This result demonstrates that minority people who commute to the suburbs are less prominent than white commuters to the cities.

However, the association between nighttime segregation and exposure levels is not statistically significant in other racial groups, in contrast to white people, although its coefficient has the sign expected from the hypotheses (i.e., positive association between nighttime segregation and exposure levels). Possible reasons for this result may be that traffic-related PM_2.5_ concentrations are spatially less variable at night or that fewer people were near high-traffic roadways at night than during the daytime, which could make it difficult to detect a significant relationship. Another possible reason is that the residential (nighttime) clustering of each minority group is not as strong as that of white people living in outer-ring suburbs or exurbs, or their clustering is not well detected by the individual-level measure of segregation due to the fewer number of samples from minority groups. Additional research is needed to further investigate the effect of nighttime segregation on exposure to traffic-related PM_2.5_. 

**Figure 4:**
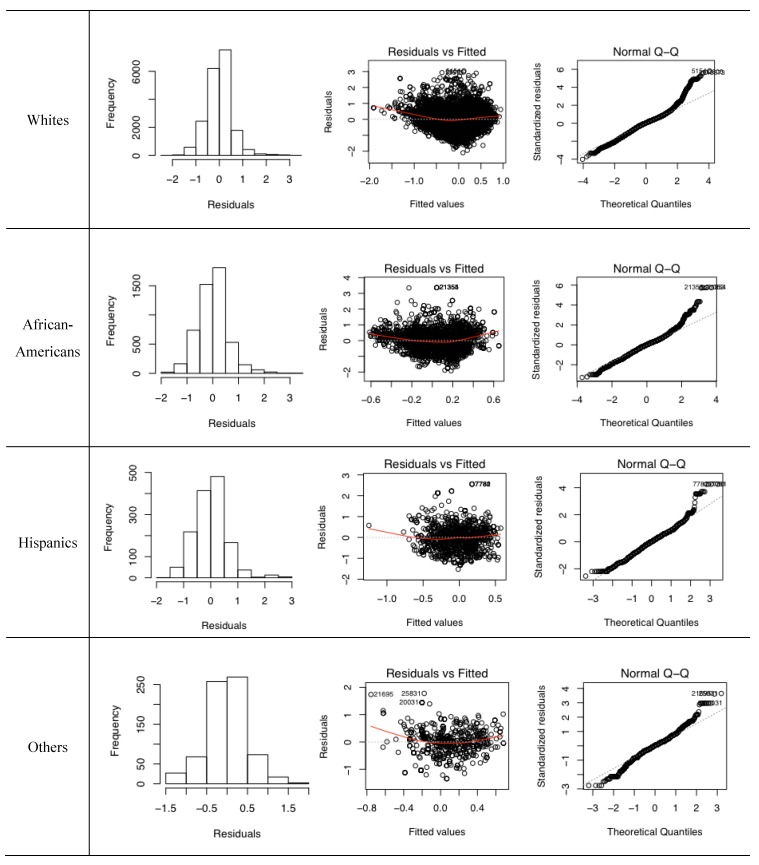
Diagnostic plots of multiple linear regression models with log-transformed variables.

The assumptions of normality and equality of variance are re-evaluated with the log-transformed variables. The diagnostic results suggest that all four regression models satisfy the assumptions (Figure 4). The regression outputs are robust because the MAUP and UGCoP, which are the sources of statistical biases, are addressed by using individual-level variables that are created by considering daily mobility.

**Figure 5:**
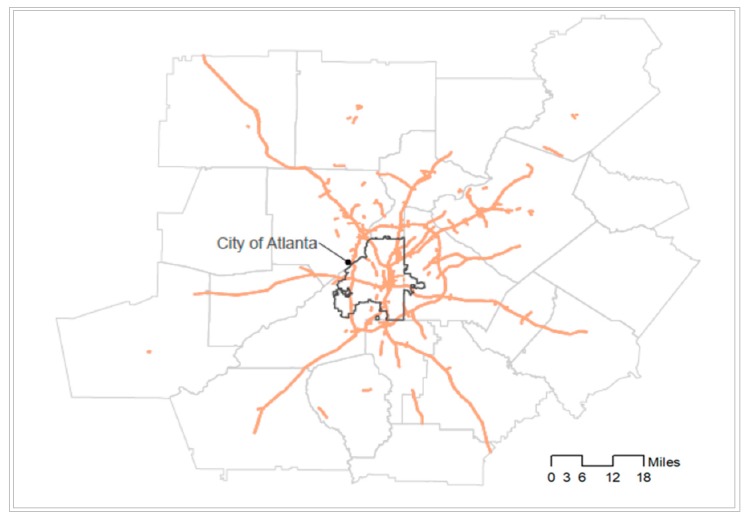
Two hundred-meter buffers around major roads (Note: Individual activity locations are not visualized together on this map to ensure data confidentiality).

To further examine racial differences in exposure to traffic-related PM_2.5_, this study performs a buffer analysis. It investigates what percentage of each racial group spends time near major roads and how that changes over the course of a day. Buffers within 200 meters from major roads are created using GIS (Figure 5) and then overlaid with individual locations in each time period of a day.

The percentage of each racial group near major roadways for each time period is calculated by dividing the number of near-road individuals of each racial group for that time period by the total number of that racial group. The result shows that during the daytime (6 a.m.–6 p.m.), a greater number of people spent their time close to the major roads than at night (6 p.m.–6 a.m.), and this temporal pattern is present for all racial groups (Table 4). However, the percentage of near-road individuals is highest in African-Americans at all times of day. For example, at 12–3 p.m., about 24% of African-Americans stayed near high-traffic roads, while roughly 17% of whites/Hispanics and 18% of Others did so. At 9 p.m.–3 a.m., it was only African-Americans whose percentage of the near-road population exceeded 10%.

## 4. Discussion

This study reveals the effect of segregation on exposure to traffic-related air pollution using individual-level, fine-scale spatiotemporal data, and methods. By considering people’s daily movement patterns and spatiotemporal distributions of racial groups, it finds that the association between segregation and exposure to traffic-related air pollution differs by race and time of day. This finding provides a new insight into the effect of segregation that occurs in non-residential contexts on environmental disparities. During the daytime, people are more integrated for work in high-traffic areas, and consequently, all racial groups share similarly high levels of traffic-related air pollution in these areas. It indicates that residence-based exposure assessments may underestimate white people’s total daily exposure to traffic-related air pollution [33]. At night, if white people are more segregated (i.e., higher levels of residential segregation), they are more likely to experience lower levels of near-road exposure. This suggests that white people specifically benefit from nighttime segregation because they are segregated into environmentally favorable areas at night. However, such a beneficial effect is not found in other racial groups. These findings indicate that although all vehicle drivers in this region, regardless of their race/ethnicity, are responsible to some degree for high levels of traffic-generated air pollution in the central cities (though white suburban/exurban commuters probably have the greater responsibility for it), white people can reduce their burden of air pollution by living in suburban/exurban areas far away from high-traffic roads, which may not always be the case for other racial groups.

This study argues that the uneven spatiotemporal distribution of racial groups and high levels of traffic-related air pollution in this region may be closely related to its public transit system. Many public transportation systems in the U.S. are strongly tied to long-standing racial discrimination [34]. The Metropolitan Atlanta Rapid Transit Authority (MARTA) system in the Atlanta metropolitan area was also developed and has been maintained based on a long history of racial and ethnic tensions. The transit lines were built to prevent people of color from reaching white-flight neighborhoods in suburbs and exurbs. 

For some people, however, public transit is not a choice but a necessity to reach work, grocery stores, health care, leisure activity places, or other places they want/need to go [35]. In this region, African-American and Latino workers are roughly five times as likely as white workers to rely on public transit to commute due to lack of a private vehicle [36]. This suggests that a number of minority people, particularly African-Americans who do not own a private vehicle, may have to live, work, shop, and undertake social and recreational activities within areas reached by the transit lines. Such limited mobility, along with racial discrimination in housing, is likely to entrap racial minorities in the inner cities or inner-ring suburbs with high-traffic volumes both during the day and at night. This conclusion agrees well with Krivo et al.’s [37] finding that the mobility of African Americans and Latinos or the poor is usually restricted into their poor residential neighborhood or non-residential areas that are similar in environmental quality to their residential neighborhood due to discrimination and social, economic, and racial constraints. According to the ARC’s annual survey, about 30% of respondents said that one of the top problems that the metro Atlanta region faces is the limited transit that renders it difficult for them to reach necessary or desired places, and nearly half of respondents said that the most ideal long-term solution to reduce traffic is to expand the regional public transit. This survey result supports the findings of this study. 

This study has some limitations that need to be addressed in future studies. First, the travel-activity diary data used for deriving the *i*-STP index values and individual exposure metrics only contains weekday movement patterns. Weekend travel patterns might be quite different because many people do not work during weekends and instead spend more time on recreational activities. Therefore, the findings may not fully reveal the disparity in exposure to traffic-related air pollution related to leisure activity places. It has been found that different racial groups tend to undertake recreational activities at different places [4,38,39,40]. If data on weekend travel patterns were to become available, it would allow us to understand how recreational activity places visited by different racial groups may further differentiate the racial disparities in exposure to environmental stressors. 

Second, the effect of segregation while traveling or commuting may not be well explained in this study because the samples are spatiotemporally not dense enough to examine the segregation of people along roadways. In addition, it has not been fully discussed whether we can say that people from different racial groups are integrated while traveling if they share the same road at the same time but are inside of their private vehicles. More discussion would be needed to clearly define what segregation while traveling means and determine how to quantify it. Quantifying racial segregation resulting from the use of different travel modes is an interesting future research topic and would provide another important insight into unequal exposure to traffic-related air pollution. Third, future research needs to look into both indoor and outdoor environments in which different social groups spend their time. Socially marginalized groups tend to occupy less favorable indoor environments than others do. Poor-quality houses are more likely to have inadequate ventilation that makes particles stay within the houses. Fourth, future research should consider the inhalation rate that may differ by the intensity of physical activity, body weight, age, or gender to estimate personal exposure levels more accurately. 

Lastly, this study does not consider spatial regression models that account for spatial autocorrelation because taking into account the complex spatial structures generated by movement data in regression models is technically very challenging. It also may not be necessary for several reasons. First, because people move during a day, there is no single fixed location from which spatial observations can be obtained for a spatial regression model, unlike residence-based approaches. The dependent variable (i.e., individuals’ total exposure levels) is generated by summing up all exposure values at multiple locations throughout the day. Second, each independent variable (daytime and nighttime segregation) has a unique spatial pattern because they are generated at different locations. These various spatial patterns may have canceled out the clustering of values among the independent variables, correcting for potential spatial dependence in a regression model. Lastly, existing spatial regression methods (e.g., spatial lag models and spatial error models) generally use a single spatial weight matrix for one regression model to address spatial dependence in the dependent variable or the error term. If we would like to consider spatial dependencies in multiple independent variables generated at different locations, we may need multiple different spatial weight matrices for a regression model, which is not possible with existing methods. Examining this interesting spatial structure in movement data remains an important area of ongoing research [41]. 

## 5. Conclusions

This study advances an understanding of the geographies of racial disparities in environmental exposure by considering dynamic population distribution and daily mobility. The findings show how non-residential segregation and uneven distribution of racial groups during the daytime contribute to the disparities in exposure to traffic-related PM_2.5_. It also shows the great potential for individual-level dynamic assessments of urban disparities and environmental injustice. It calls into question past studies’ residence-based air pollution exposure metrics and measures of residential segregation and highlights the importance of considering human mobility in environmental justice studies. The individual-level, mobility-based measures of segregation and exposure estimates deployed in this study addresses the MAUP and UGCoP––the problems that have made previous studies’ findings less reliable. 

Although the present study focuses on traffic-related PM_2.5_ and the Atlanta metropolitan area, the methods used in this study are widely applicable to other air pollutants and other areas where a similar kind of data (e.g., travel-activity diaries) is available, including the greater Chicago area and southern California region. To the best of our knowledge, this is the first study to reveal the role of spatiotemporally uneven population distribution in unequal exposure to traffic-related PM_2.5_. It offers important insights into the development of more effective policies for addressing environmental health disparities. Given that racial disparities in exposure to air pollution are the result of systematic discrimination in housing, public transportation, and environmental policies that may impose disadvantages on racial minorities, this study suggests that policies for improving the regional transit system should be implemented together with policies for residential mixes in order to reduce traffic-related air pollution and to equally distribute the benefit of clean air and the burden of air pollution to all racial groups. As the findings reveal that all racial groups who work/spend time in the central cities are likely to be exposed to very high traffic-related PM_2.5_, the effort toward more extensive and equitable public transit systems would contribute to mitigating everyone’s exposure to traffic-related air pollution and health risk as well as achieving environmental justice and health equity. 

## Figures and Tables

**Table 1:** Racial differences in exposure to traffic-related PM_2.5_.

Racial Groups	Difference of Means	*p*-Value
African-Americans–Whites	0.1504 ***	<0.001
Hispanics–Whites	0.0563	0.2758
Others–Whites	0.1636 ***	<0.001
Hispanics–African-Americans	−0.0941*	0.0306
Others–African-Americans	0.0132	0.9907
Others–Hispanics	0.1073	0.1543

*** *p* ≤ 0.001; * *p* ≤ 0.05.

**Table 2:** Descriptive statistics for dependent and independent variables.

		Whites	African-Americans	Hispanics	Others
**Exposure levels**	Mean	1.18	1.33	1.24	1.35
	Median	0.97	1.05	0.98	1.13
	Max	26.93	30.19	15.36	35.30
	Min	0.14	0.17	0.21	0.22
	S.D.	1.05	1.43	1.18	1.71
**log(daytime segregation)**	Mean	0.53	0.57	0.56	0.54
	Median	0.53	0.53	0.56	0.53
	Max	1.30	1.13	1.42	1.10
	Min	0.13	0.13	0.17	0.19
	S.D.	0.14	0.20	0.16	0.15
**log(nighttime segregation)**	Mean	0.70	0.79	0.78	0.73
	Median	0.67	0.77	0.72	0.69
	Max	1.77	1.41	1.49	1.39
	Min	0.24	0.18	0.31	0.32
	S.D.	0.17	0.22	0.20	0.18
**Age**	Mean	40.10	38.60	33.55	37.44
	Median	44.00	41.00	36.00	41.00
	Max	93.00	87.00	87.00	87.00
	Min	0.00	0.00	0.00	0.00
	S.D.	21.29	20.40	19.87	20.12
**Gender (male:0; female:1)**	Mean	0.53	0.60	0.49	0.50
**Income dummy 1**	Mean	0.10	0.28	0.23	0.15
**Income dummy 2**	Mean	0.18	0.30	0.21	0.18
**Education dummy 1**	Mean	0.27	0.27	0.40	0.28
**Education dummy 2**	Mean	0.30	0.40	0.27	0.22

**Table 3:** Race-specific regression analysis results.

Variables	Whites	African-Americans	Hispanics	Others
Main independent variables				
log(daytime segregation)	−1.836 ***	−0.823 ***	−1.484 ***	−1.273 ***
log(nighttime segregation)	−0.338 ***	0.040	0.113	−0.272
Control variables				
Age	−0.000	0.000 ***	−0.000	−0.000
Gender (male:0; female:1)	−0.030 ***	0.018	−0.065 *	−0.006
Income dummy 1	−0.168 ***	0.063 **	0.215 ***	0.194 **
Income dummy 2	−0.082 ***	0.053 *	−0.058	−0.077
Education dummy 1	−0.212 ***	−0.281 ***	−0.151 ***	−0.106 *
Education dummy 2	−0.180 ***	−0.017	0.094 *	−0.223 ***

*** *p* ≤ 0.001; ** *p* ≤ 0.01; * *p* ≤ 0.05.

**Table 4:** The percentage (%) of the near-road population at different times of day (by race).

Race	The Percentage (%) of the Near-Road Population (Within 200 Meters from Major Roads) at Different Times of Day						
	3–6 a.m.	6–9 a.m.	9 a.m.–12 p.m.	12–3 p.m.	3–6 p.m.	6–9 p.m.	9 p.m.–3 a.m.
Whites	4.6	10.5	16.2	17.1	13.7	10.2	6.4
African-Americans	9.7	17.6	23.1	23.7	22.1	16.1	12.2
Hispanics	6.6	10.3	15.9	17.0	14.2	11.5	7.6
Otherss	7.6	15.0	18.8	18.2	19.8	15.4	9.9
